# Identification of Costimulatory Molecule–Related lncRNAs Associated With Gastric Carcinoma Progression: Evidence From Bioinformatics Analysis and Cell Experiments

**DOI:** 10.3389/fgene.2022.950222

**Published:** 2022-08-05

**Authors:** Zhenhua Yin, Yating Qiao, Jianping Shi, Limei Bu, Li Ao, Wenqing Tang, Xiaolan Lu

**Affiliations:** ^1^ Department of Digestive, Shanghai Pudong Hospital, Fudan University Pudong Medical Center, Shanghai, China; ^2^ Department of Gastrointestinal Surgery, Affiliated Hospital of Hebei University, Baoding, China; ^3^ Department of Gslastroenterology and Hepatology, Shanghai Institute of Liver Disease, Zhongshan Hospital, Fudan University, Shanghai, China

**Keywords:** costimulatory molecule, lncRNAs, AP000695.2, gastric, cancer

## Abstract

Costimulatory molecules (CMGs) play essential roles in multiple cancers. However, lncRNAs regulating costimulatory molecules have not been fully explored in gastric cancer (GC). Public data of GC patients were obtained from The Cancer Genome Atlas database. R software v4.1.1, SPSS v13.0, and GraphPad Prism 8 were used to perform all the analyses. The Limma package was used for differential expression analysis. The survival package was used for patient prognosis analysis. The gene set enrichment analysis (GSEA), gene ontology (GO), and the Kyoto encyclopedia of genes and genomes (KEGG) analysis were used for pathway enrichment analysis. qRT-PCR was used to detect the RNA level of target lncRNA. CCK-8 and colony formation assay were used to assess the proliferation ability of GC cells. The transwell assay was used to evaluate the invasion and migration ability of GC cells. We first identified CMG-related lncRNAs (CMLs) through co-expression analysis. Then, an eight-CML-based signature was constructed to predict patient overall survival (OS), which showed satisfactory predictive efficiency (the training cohort: 1-year AUC = 0.764, 3-year AUC = 0.810, 5-year AUC = 0.840; the validation cohort: 1-year AUC = 0.661, 3-year AUC = 0.718, 5-year AUC = 0.822). The patients in the high-risk group tend to have a worse prognosis. GSEA showed that epithelial–mesenchymal transition, KRAS signaling, and angiogenesis were aberrantly activated in high-risk patients. GO and KEGG analyses indicated that the biological difference between high- and low-risk patients was mainly enriched in the extracellular matrix. Immune infiltration analysis showed that macrophages (M1 and M2), dendritic cells, monocytes, Tregs, and T regulatory cells were positively correlated with the risk scores, partly responsible for the worsening OS of high-risk patients. Finally, lncRNA AP000695.2 was selected for further experiments. The result showed that AP000695.2 was upregulated in GC cell lines and could facilitate the proliferation, invasion, and migration of GC cells. In summary, this study established an effective prognosis model based on eight CMLs, which would be helpful for further therapy options for cancer. Also, we found that AP000695.2 could promote GC cell malignant phenotype, making it an underlying therapy target in GC.

## Introduction

Gastric cancer (GC) is the fourth most common malignant tumor globally, resulting in over 1.2 million new cases and 900,000 cancer-related deaths per year (Smyth et al., 2020). Epidemiological research results demonstrated that the incidence of GC in young people is gradually increasing, with the characteristics of high morbidity, metastasis, and mortality (Sitarz et al., 2018). Meanwhile, due to the lack of specific signs of early GC, most patients have already developed advanced GC with poor prognosis at their first diagnosis (Song et al., 2017). Recently, the combined application of chemotherapy and targeted therapy has significantly prolonged the overall survival of patients with advanced GC (Stewart et al., 2020). Since the GC has specific precancerous lesions, early screening and monitoring of patients with these lesions can significantly reduce the incidence and mortality of GC, especially in the countries with high incidence like Japan and South Korea (Wei et al., 2020). Some mechanisms of GC have been explored, leading to the application of primary and secondary prevention, including a healthy lifestyle and the eradication of *Helicobacter pylori* (Correa, 2013). Therefore, it is still urgent to explore early diagnosis markers to improve the early diagnosis rate and extend the survival time of GC patients.

Costimulatory molecules are cell surface molecules and their ligands that provide costimulatory signals for the complete activation of T cells or B cells (Podojil and Miller, 2009). T lymphocytes express CD28, and antigen-presenting cells express CD80 and B7-2 CD86 (Pasquini et al., 1997). A previous study has discovered the potential application of the cell costimulatory molecule OX40 and its homologous ligand OX40L in autoimmune diseases and cancer immunotherapy (Karimi et al., 2013). In addition, when combined with other therapy, including anti-PD-1, anti-CTLA-4 therapy, cytokines, chemotherapy, or radiotherapy, the antitumor activity of anti-OX40 treatment will be further enhanced (Wei et al., 2018). With the rapid development in cancer biology, many molecular targeted drugs have demonstrated significant antitumor activity in various tumors (Tamada and Chen, 2000). Immunotherapy, as a new type of anticancer therapy, is applied to identify and eliminate malignant tumors by using immune monitoring to inhibit the development of tumors (Mueller, 2000). The success of immune checkpoint inhibitors might depend on the deep understanding of immunosuppressive conditions in the human immune system (Schietinger and Greenberg, 2014). The naive T cells need two signals to be active. The first signal is the recognition of T cell receptors to a specific antigen, and the second is the unspecific costimulatory signal, which was essential for T cell attack (Bluestone, 1995). Costimulatory molecules are classified as B7-CD28 and TNF family members. Long noncoding RNA (lncRNA) can perform diverse biological functions with more than 200 nucleotides (Ferrè et al., 2016). Some studies have investigated the role of lncRNAs in regulating costimulatory molecules. For example, Zhao et al. (2019) revealed that lncRNA SNHG14 could regulate the PD-1/PD-L1 checkpoint through a positive feedback loop miR-5590-3p/ZEB1 axis, further promoting diffuse large B cell lymphoma progression and immune evasion. Also, Zhang et al. (2020) showed that lncRNA GATA3-AS1 could facilitate tumor progression and immune escape in triple-negative breast cancer through destabilization of GATA3 but stabilization of PD-L1. Therefore, there is a need for full-scale investigations of lncRNAs participating in regulating costimulatory molecules in GC.

Nowadays, the rapid development of bioinformatics technology brings great convenience for researchers. This study identified eight prognosis-related CMLs AC027117.2, AC016737.1, LINC01614, AC147067.2, AP000695.2, LINC01094, HAGLR, and AL365181.3, which might play an important role in GC. Based on these CMLs, an effective prognosis model was constructed to predict GC patient prognosis, which showed satisfactory prediction efficiency. Following this, we performed pathway enrichment and immune infiltration analysis to explore the biological difference between high- and low-risk patients. Finally, CML AP000695.2 was selected for further analysis for its correlation with T and N stages. *In vitro* experiments indicated that AP000695.2 was upregulated in GC cells and could promote GC progression.

## Materials and Methods

### Data Acquisition and Preprocessing

The Cancer Genome Atlas database, (TCGA, https://portal.gdc.cancer.gov/), a project supervised by the National Cancer Institute and the National Human Genome Research Institute, aims to apply the high-throughput genome analysis technology and help people obtain a better understanding of cancer, thereby improving the abilities of cancer prevention, diagnosis, and treatment. In this study, the transcriptome data of GC patients (TCGA-STAD) and their clinical information were downloaded from the TCGA dataset, which involved 32 normal samples and 375 tumor samples. In addition, 59 costimulatory molecules (CMGs) were finally identified based on previous studies (Croft et al., 2013; Schildberg et al., 2016). The Homo_sapiens.GRCh38.gtf reference file was used for probe annotation. Based on the co-expression analysis, CMG-related lncRNAs (CMLs) were identified, meeting the Pearson correlation coefficient (|cor| > 0.30, *p* < 0.05) with the costimulatory molecules. The limma package in R software was applied to perform the differential expression analysis on GC samples compared with normal samples.

### Construction and Validation of the Prognostic Costimulatory Molecule Risk-Scoring Model

In addition, to further establish and verify the CML-based prognosis prediction model, GC patients with complete follow-up information in TCGA-STAD are randomly divided into training and validation cohorts. Next, the survival package in R software was applied in univariate Cox regression to identify prognosis-related CMLs. The Lasso regression analysis was used to reduce the influence of collinearity. We finally constructed the CMG-based prognostic prediction model by performing multivariate Cox regression. The risk score of each patient was calculated with the formula of “Risk score = CoefA * GeneA + CoefB * GeneB + … + CoefN * GeneN”. The Kaplan–Meier survival analysis and receiver operating characteristic (ROC) curves were used to evaluate the stability of the prognostic model. The area under the ROC curve (AUC) value > 0.65 was considered to have a good predictive value, and > 0.8 was regarded to have an excellent predictive value.

### Clinical Correlation and Pathway Enrichment

The clinical data of GC patients, including age, gender, tumor grade, and pathological stage, were downloaded from the TCGA database. Then, the relationship between prognosis-related lncRNAs, risk score, and clinical characteristics was evaluated by a box plot. We also performed univariate and multivariate Cox regression analyses to explore whether the prognostic model is independent of other traditional clinical features. In addition, the nomogram, which involved the clinical features and risk scores, was constructed based on TCGA-STAD cohorts. The calibration plot was drawn to evaluate the predicted probability and fitness of the nomogram. Gene Ontology (GO) and Kyoto Encyclopedia of Genes and Genomes (KEGG) enrichment analyses were performed by ClusterProfiler packages in R software to explore the underlying biological difference between high- and low-risk patients (Yu et al., 2012). Gene set enrichment analysis (GSEA) was used to explore the correlation between risk scores and cancer pathways and the reference gene set was the “Hallmark” (Subramanian et al., 2005).

### The Immune Infiltration Analysis

The CIBERSORT algorithm was applied to analyze the RNA-seq data of GC patients to evaluate the relative proportions of 22 immune infiltrating cells in low- and high-risk groups (Chen et al., 2018). The Spearman correlation analysis was performed on gene expression and immune cell distribution, and *p* < 0.05 was considered statistically different. ESTIMATE in R software was used to estimate the proportion of the cells in the tumor microenvironment, shown in ImmuneScore, StromalScore, and EstimateScore forms.

### Immunotherapy and Drug Sensitivity Analyses

Tumor Immune Dysfunction and Exclusion (TIDE) analysis was performed to explore the underlying response rate difference of GC patients on immunotherapy (Jiang et al., 2018). The drug sensitivity analysis was conducted based on the Genomics of Drug Sensitivity in Cancer (GDSC) database (Yang et al., 2013).

### Cell Lines and Quantitative Real-Time PCR

Human normal gastric mucosal cells (GES-1) and gastric cancer cells (MGC-803, BGC-823, AGS, HGC-27) were laboratory stock. Total RNA was extracted using a RNA simple total RNA kit (Invitrogen). The SYBR Green PCR system was used to perform Quantitative Real-Time PCR (qRT-PCR). The shRNA sequences targeting for the human ENSG00000233818 (AP000695.2) longest transcript were designed with Invitrogen online design software (BLOCK-iTTM RNAi Designer). The primers used were as follows: AP000695.2, forward primer: 5′-ACC​CAG​CCA​ACT​TGT​CAT​GT-3′, reverse primer: 5′-ACA​TCT​TCC​TGC​ACT​GCC​AA-3′, GAPDH: 5′-GGA​GCG​AGA​TCC​CTC​CAA​AAT-3′, reverse primer: 5′-GGC​TGT​TGT​CAT​ACT​TCT​CAT​GG-3′.

### Cell Transfection

Cell transfection was performed using Lipofectamine 2000 following the standard process (AGS and HGC-27 cells). The shRNA primers used were as follows:

### Cell Proliferation Assay

CCK-8 and colony formation assays were used to evaluate the proliferation ability of GC cells. Briefly, for the CCK-8 assay, cells were seeded into a 96-well plate and added with the CCK-8 reagent according to the manufacturer’s instructions. At the set time (0, 24, 48, and 72 h), cell proliferation ability was detected by measuring the absorbance at 460 nm. For the colony formation assay, cells were seeded into a six-plates well with 800 cells per well. Then, the cells were cultured routinely for 14 days (the medium was replaced every 4 days). Finally, cell clones were fixed and stained with crystal violet.

### Transwell Assay

Transwell assay was performed to assess the invasion and migration ability of GC cells using 24-well transwell chambers. Briefly, cells with the serum-free medium were added into the upper chambers with or without Matrigel. Lower chambers were added with a medium (600 μl) containing 10% serum. After 24 h, cells were fixed and stained.

### Data Analysis

**FIGURE 1:**
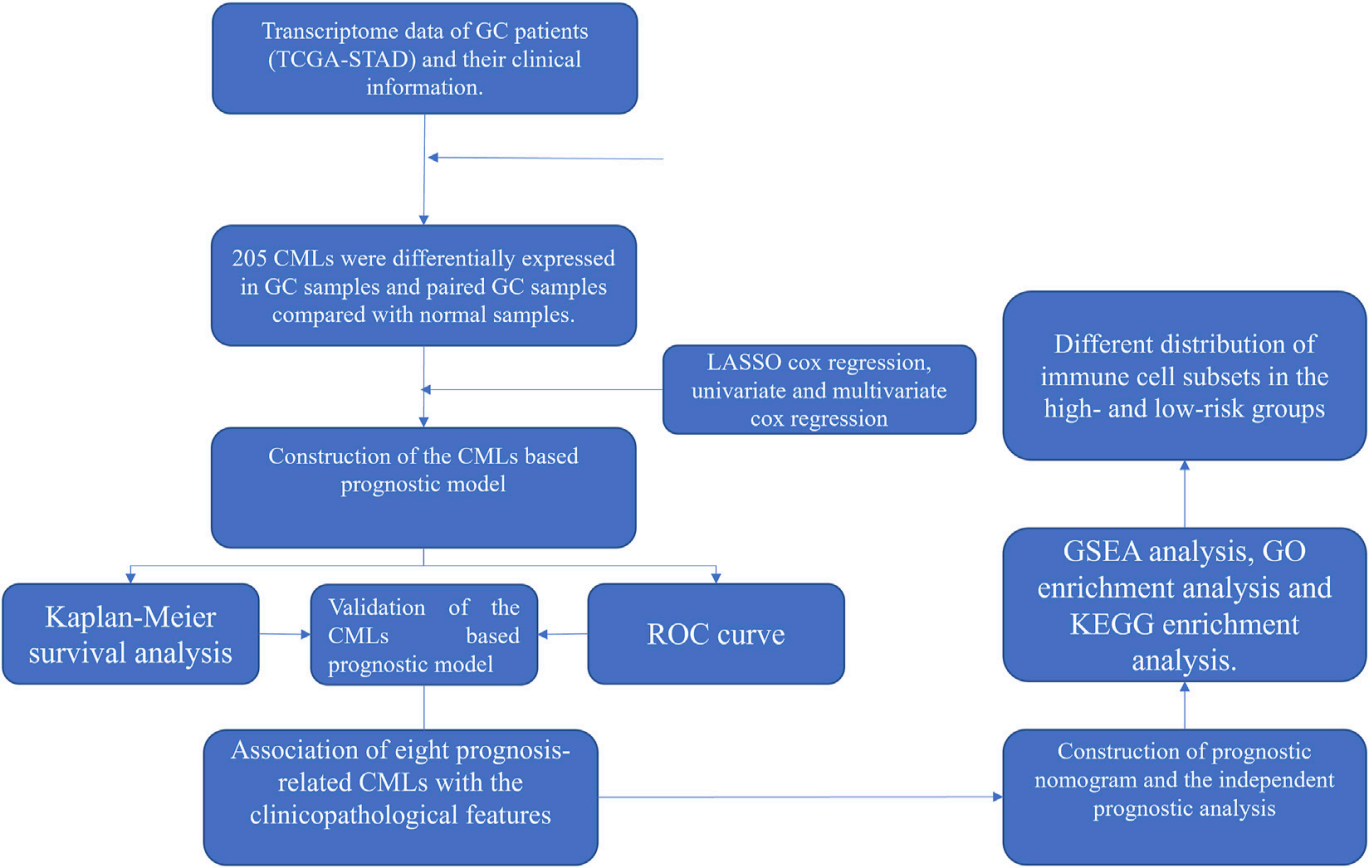
Flowchart of the whole study.

All the analyses were performed in R software v4.1.1, SPSS v13.0, and GraphPad Prism 8. The *p*-value was two-sided and < 0.05 was regarded as statistically significant. Student’s *t*-test was used to compare the difference between the two groups. The workflow is shown in Figure 1 *.*

## Results

### Identification of CMG-Related lncRNAs and Differential Expression Analysis

**FIGURE 2:**
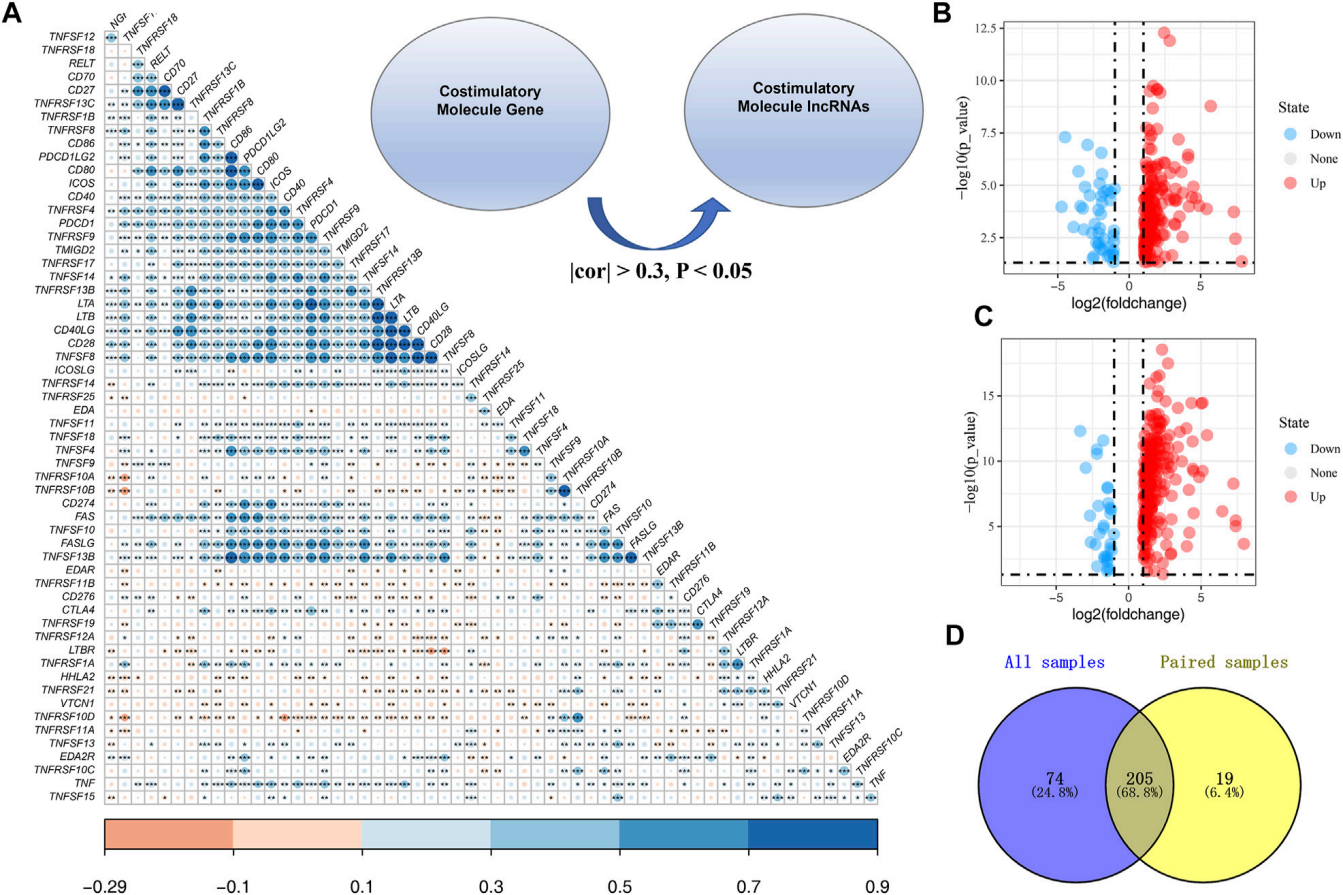
Identification of costimulatory molecule–related lncRNAs. **(A)**: Overview of CMGs in the TCGA cohort; Co-expression analysis was performed to identify costimulatory molecule–related lncRNAs with the Pearson correlation coefficient of |cor| > 0.3 and *p* < 0.05 with CMGs; **(B)**: Differential CML analysis between all normal and GC tissues; **(C)**: Differential CML analysis between paired normal and GC tissues; **(D)**: Venn diagrams showed that 205 CMLs were differentially expressed in GC samples and paired GC samples compared with normal samples.

The workflow of the whole study is shown in Figure 1. We first performed the co-expression analysis to identify CMLs with the Pearson correlation coefficient of |cor| > 0.3 and *p* < 0.05 with CMGs (Figure 2A). Based on these CMLs, we performed a differentially expressed analysis between normal and GC tissues. A total of 279 and 224 differentially expressed lncRNAs were identified from all GC samples and paired GC samples compared with normal samples, respectively (Figures 2B,C). As is shown in Figure 2D, 205 CMLs were differentially expressed in GC samples and paired GC samples compared with normal samples, which were selected for further analysis.

### Construction and Validation of the CMGs-Related lncRNAs Based Prognostic Model

**FIGURE 3:**
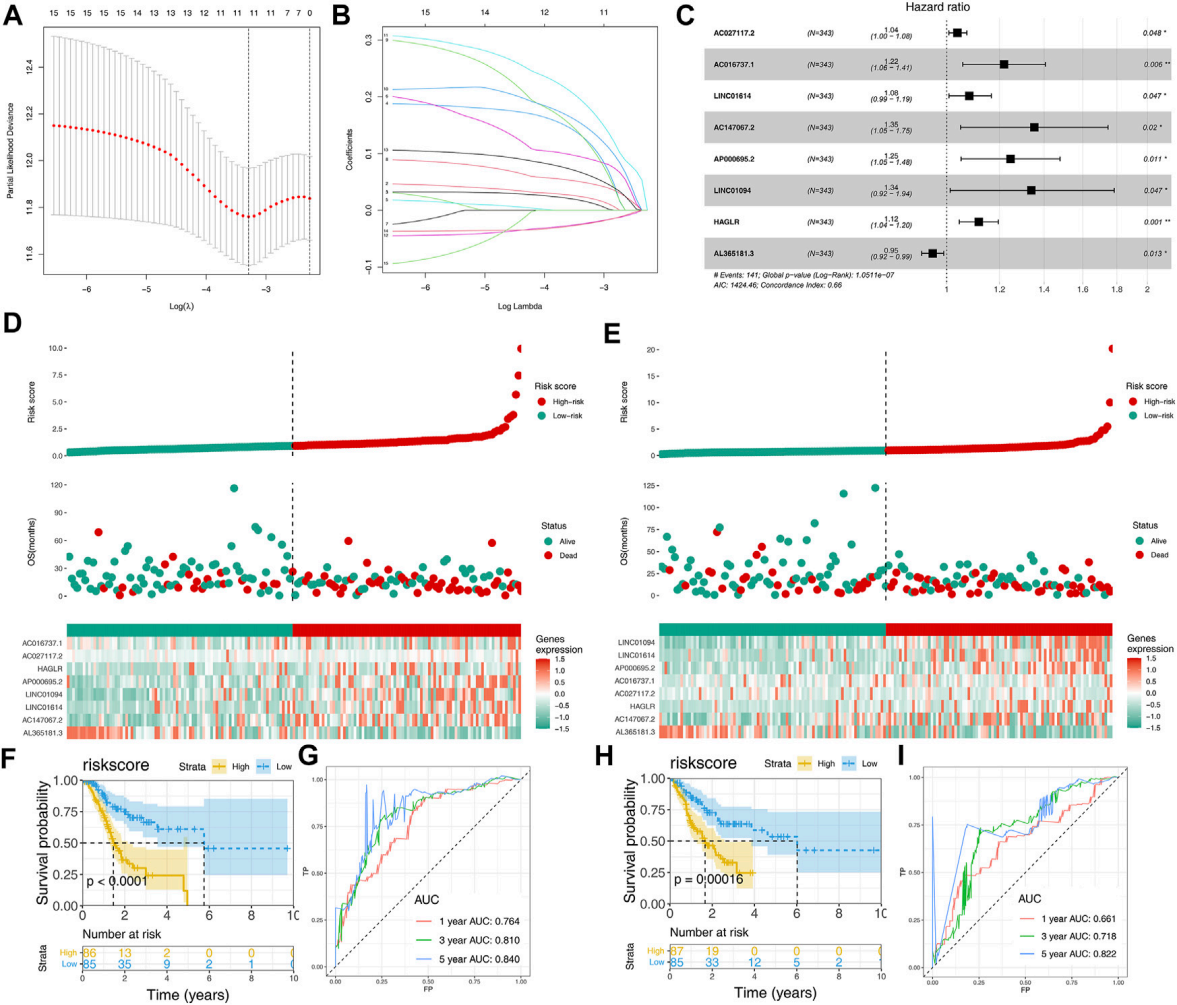
Construction and validation of the CML-based prognosis model. **(A,B)**: Lasso regression analysis was performed to conduct dimension reduction; **(C)**: Multivariate Cox regression analysis was performed to construct a prognostic model with *p* < 0.05 and the model genes include AC027117.2, AC016737.1, LINC01614, AC147067.2, AP000695.2, LINC01094, HA,GLR and AL365181.3; **(D)**: Riskplot of patients in the training cohort; **(E)**: Riskplot of patients in the validation cohort; **(F,G)**: Kaplan–Meier survival and ROC curves in the training cohort; **(H,I)**: Kaplan–Meier survival and ROC curves in the validation cohort.

A total of 47 prognosis-related CMLs were identified by using the univariate cox regression analysis for their significant association with the overall survival (OS) of GC patients (Table 1). Furthermore, we performed the LASSO Cox regression analysis based on the aforementioned prognostic-related genes, and the optimal model was constructed with the least parameters when the lambda was minimum (Figures 3A,B). Finally, eight CMLs AC027117.2, AC016737.1, LINC01614, AC147067.2, AP000695.2, LINC01094, HAGLR, and AL365181.3 were identified to construct a prognostic model through multivariate Cox regression (Figure 3C). Each patient was assigned a risk score with the formula of “Risk score = 0.037 * AC027117.2 + 0.198 * AC016737.1 + 0.077 * LINC01614 + 0.303 * AC147067.2 + 0.221 * AP000695.2 + 0.292 * LINC01094 + 0.111 * HAGLR + −0.048 * AL365181.3”. The patients were then randomly divided into training and validation cohorts at the ratio of 1:1. According to the median cutoff, patients were divided into low-risk and high risk groups. The result revealed that GC patients in the high-risk group had a higher overall mortality rate than the low-risk group (Figures 3D,E). Meanwhile, the Kaplan–Meier survival analysis demonstrated that patients in the low-risk group had a longer survival time compared with the high-risk group in the training cohort (Figure 3F). The ROC curve showed that our model had a satisfactory prediction efficiency (Figure 3G; 1-year AUC = 0.764, 3-year AUC = 0.810, 5-year AUC = 0.840). Similar conclusions were also observed in the validation cohort (Figures 2H,I; 1-year AUC = 0.661, 3-year AUC = 0.718, 5-year AUC = 0.822).

**TABLE 1:** Prognosis-related CMLs identified by the univariate Cox regression analysis.

ID	HR	HR.95L	HR.95H	*p* value
LINC01094	1.636	1.252	2.138	0.000
LINC01614	1.122	1.049	1.199	0.001
AP000695.1	1.373	1.137	1.658	0.001
AP000695.2	1.279	1.098	1.488	0.002
LINC02544	1.116	1.036	1.203	0.004
HAGLR	1.088	1.026	1.153	0.005
AP001528.2	1.435	1.111	1.852	0.006
AC009948.1	1.233	1.055	1.440	0.008
AC016737.1	1.225	1.053	1.424	0.008
AL365181.3	0.952	0.918	0.988	0.009
AL139393.2	1.122	1.024	1.230	0.014
TNFRSF10A-AS1	0.896	0.822	0.978	0.014
AC037198.1	1.223	1.041	1.436	0.014
AC147067.2	1.365	1.064	1.751	0.014
AC027117.2	1.046	1.008	1.087	0.018
IPO5P1	0.804	0.671	0.963	0.018
RRN3P2	1.409	1.057	1.879	0.019
AC002401.3	1.275	1.033	1.572	0.024
AC090579.1	0.590	0.373	0.933	0.024
AC006547.1	0.752	0.586	0.964	0.024
SREBF2-AS1	0.745	0.576	0.963	0.025
JPX	0.882	0.790	0.984	0.025
AC106739.1	1.212	1.023	1.437	0.026
AP001189.3	1.175	1.019	1.355	0.027
AC104041.1	1.109	1.011	1.216	0.028
AC010768.2	0.642	0.432	0.954	0.028
LINC01819	0.964	0.933	0.996	0.029
LINC00106	0.870	0.767	0.987	0.031
HOTTIP	0.884	0.789	0.991	0.035
AC110285.6	1.196	1.012	1.414	0.035
MSC-AS1	1.372	1.022	1.843	0.035
PTPRJ-AS1	0.814	0.670	0.989	0.038
MAGI2-AS3	1.195	1.009	1.415	0.039
AL135925.1	1.230	1.010	1.498	0.039
AC007255.1	0.807	0.658	0.990	0.040
AC084033.3	1.191	1.007	1.408	0.041
LINC01705	1.089	1.002	1.182	0.044
AP002954.1	1.261	1.007	1.580	0.044
LINC01978	0.860	0.742	0.996	0.044
AL589843.1	1.156	1.003	1.332	0.045
AC004596.1	0.698	0.492	0.992	0.045
PCAT19	1.252	1.005	1.561	0.045
AC116914.2	0.809	0.657	0.996	0.046
AC087286.2	1.284	1.004	1.641	0.046
AC032044.1	0.688	0.477	0.994	0.047
AC113139.1	1.195	1.002	1.425	0.047
RASSF8-AS1	1.067	1.000	1.138	0.049

### Association of Eight Prognosis-Related CMG-Related lncRNAs With the Clinicopathological Features

**FIGURE 4:**
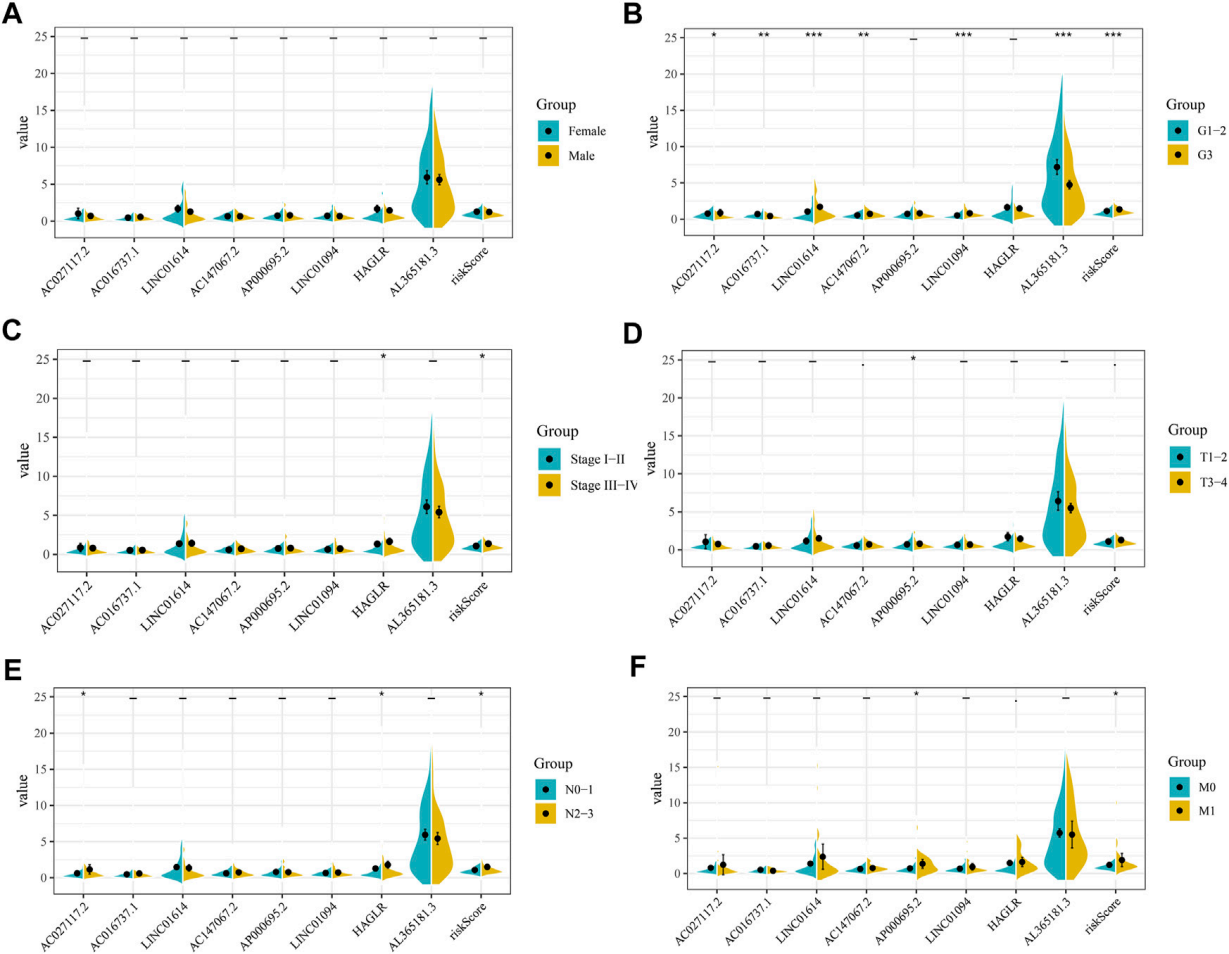
Clinical correlation of risk score and CMLs. **(A)**: Level of risk score and model CMLs in different gender patients; **(B)**: Level of risk score and model CMLs in different grade patients; **(C)**: Level of risk score and model CMLs in different stage patients; **(D)**: Level of risk score and model CMLs in different T stage patients; **(E)**: Level of risk score and model CMLs in different N stage patients; **(F)**: Level of risk score and model CMLs in different M stage patients.

Next, we evaluated the correlation between eight prognosis-related CMLs and the clinicopathological features (gender, grade, stage, and the TNM stage). As shown in Figure 4, the percentage of risk scores in grade III patients is higher than that of grade I–II patients. In addition, GC patients with higher risk scores are more inclined to be accompanied by higher M and N stages (*p* < 0.05). However, the risk score is demonstrated to have no significant differences in gender and T stage (*p* > 0.05). Further analysis showed that some prognosis-related CMLs were associated with the clinicopathological features. All eight CMLs were associated with tumor grade. AP000695.2 had a higher expression pattern in worse T and M stage patients. HAGLR was associated with patient clinical and N stages.

### Construction of Prognostic Nomogram and the Independent Prognostic Analysis

**FIGURE 5:**
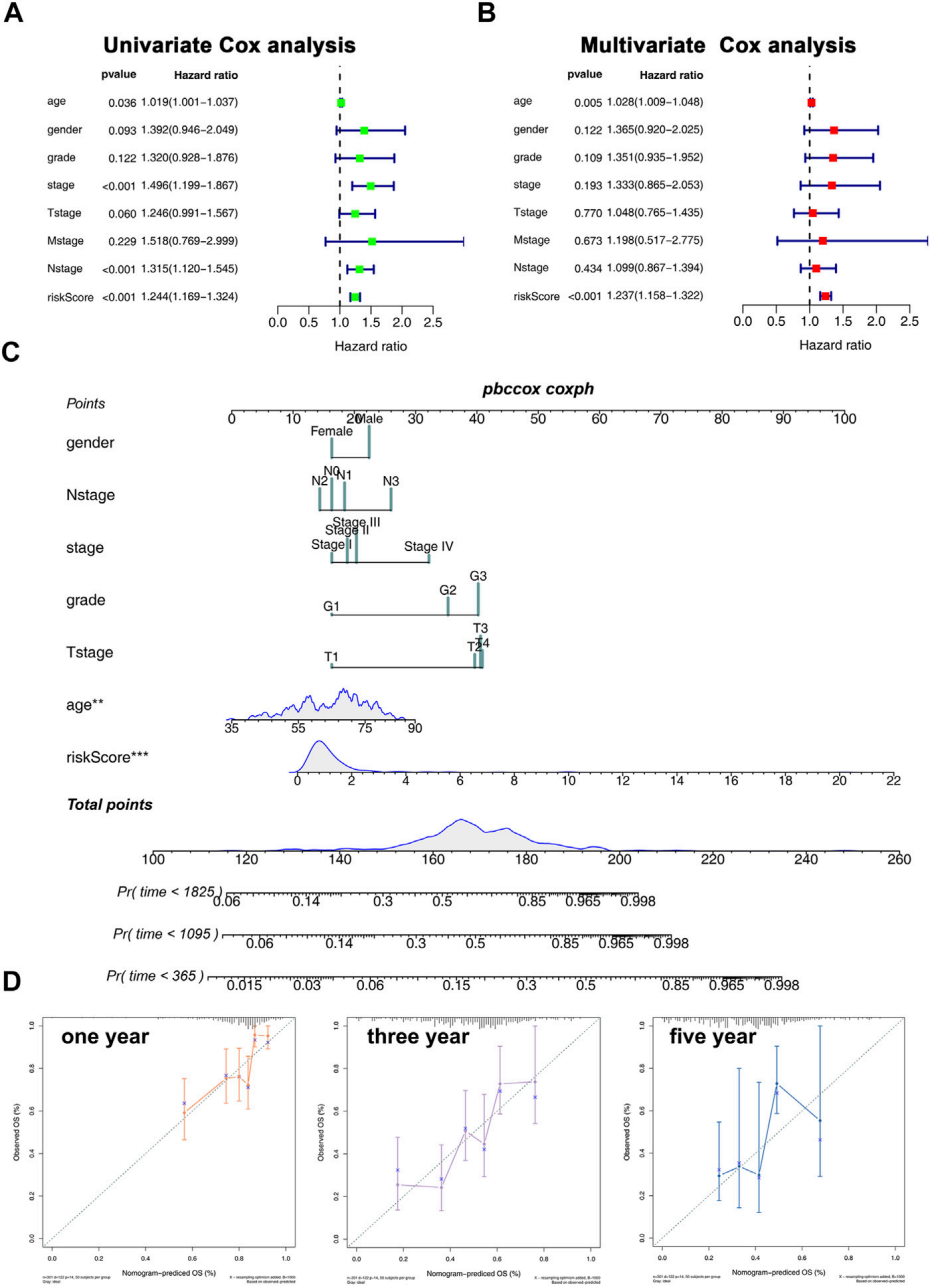
Nomogram and calibration plots. **(A,B)**: Univariate **(A)** and multivariate **(B)** analyses were performed to explore whether the association between risk core and patients prognosis is independent of clinical features; **(C)**: Nomogram was constructed based on the risk score and clinical features; **(D)**: calibration plot of 1-, 3-, and 5-year survival of nomogram predicted survival.

We then performed univariate and multivariate analyses to explore whether the association between risk score and patient prognosis is independent of clinical features. The result showed that the risk score exerts its role independently on other clinical features (Figures 5A,B). Next, a nomogram was constructed based on the clinical features and our model to better predict patient prognoses (Figure 5C). In addition, the calibration plots for the survival rate at 1, 3, and 5 years demonstrate good agreements between the nomogram predictions and actual observations (Figure 5D).

### Pathway Enrichment Analysis

**FIGURE 6:**
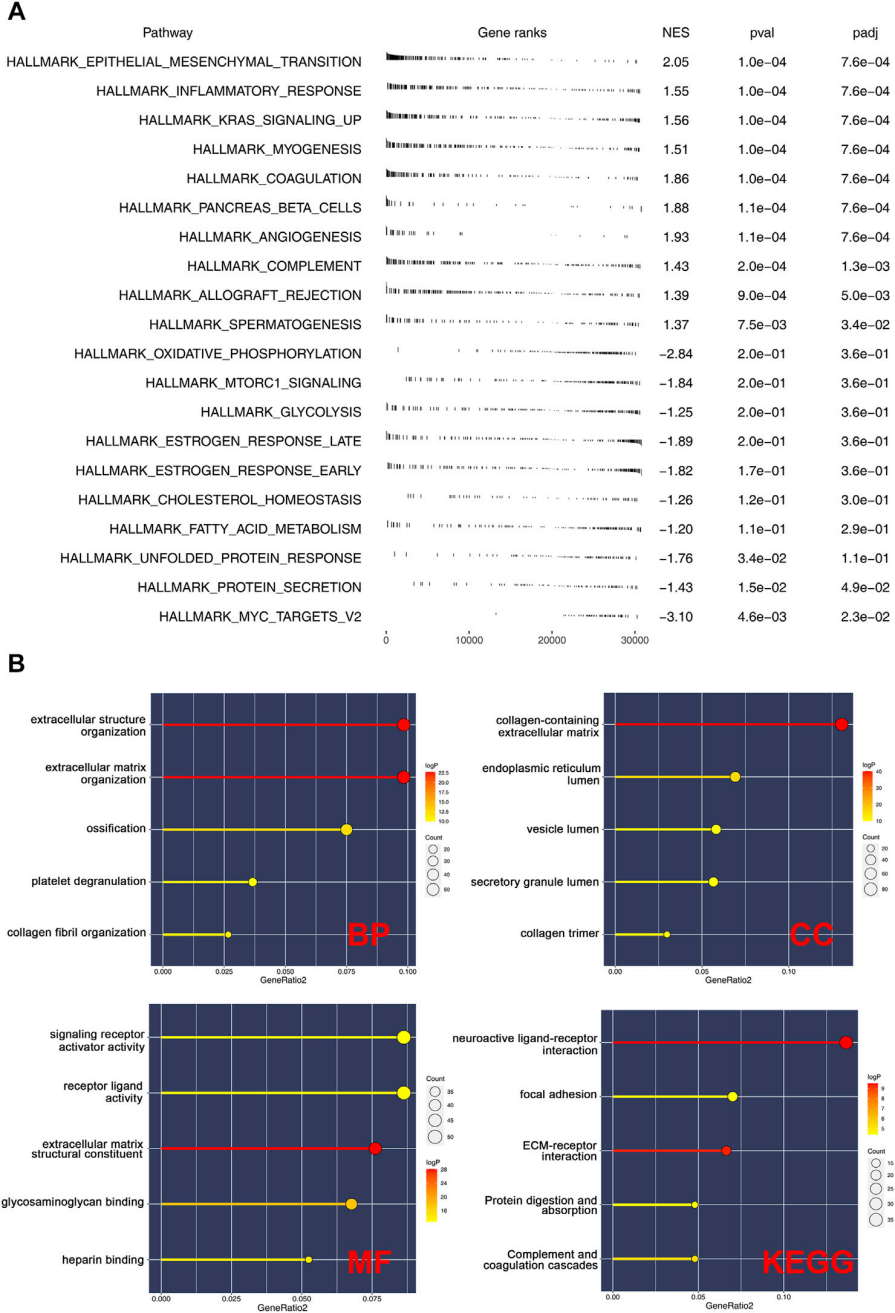
Pathway enrichment analysis of risk score. **(A)**: GSEA was performed based on the Hallmark gene set; **(B)**: GO and KEGG analyses were performed to explore the biological difference between high- and low-risk patients.

GSEA was performed to explore the biological functions of prognosis-related CMGs. The results showed that the high-risk group was significantly associated with epithelial–mesenchymal transition (EMT), inflammatory response, KRAS signaling, myogenesis, coagulation, pancreas beta cells, and angiogenesis (Figure 6A). The GO enrichment analysis showed that the biological process (BP) was mostly enriched in extracellular structure, extracellular matrix, ossification, platelet degranulation, and collagen fibril organization (Figure 6B). In cellular component (CC), the most enrichment pathway includes collagen-containing extracellular matrix, endoplasmic reticulum lumen, secretory granule lumen, vesicle lumen, and collagen trimer. The most enriched terms in molecular function (MF) include signaling receptor activator activity, receptor–ligand activity, extracellular matrix structural constituent, and glycosaminoglycan binding. The KEGG enrichment analysis was mainly enriched in neuroactive ligand–receptor interaction, focal adhesion, ECM–receptor interaction, protein digestion, absorption, and complement and coagulation cascades.

### The Risk Score Affect the Immune Microenvironment As Well As the Sensibility of Immunotherapy and Chemotherapy in Gastric Cancer

**FIGURE 7:**
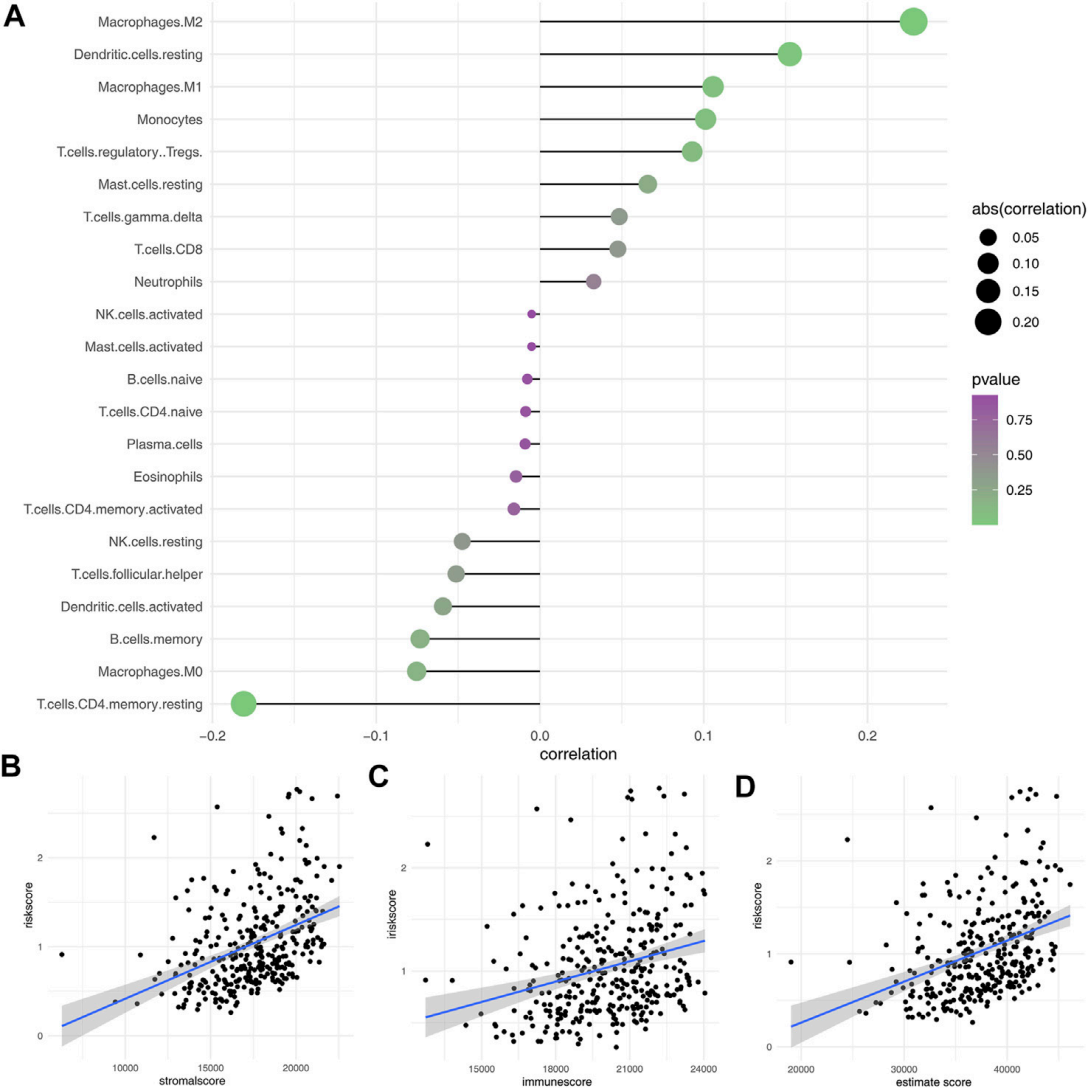
Immune infiltration analysis. **(A)**: CIBERSORT algorithm was used to evaluate the relative proportions of 22 immune infiltrating cells in low- and high-risk groups; **(B–D)**: Correlation between risk score and immune score, stromal score, and estimate score.

**FIGURE 8:**
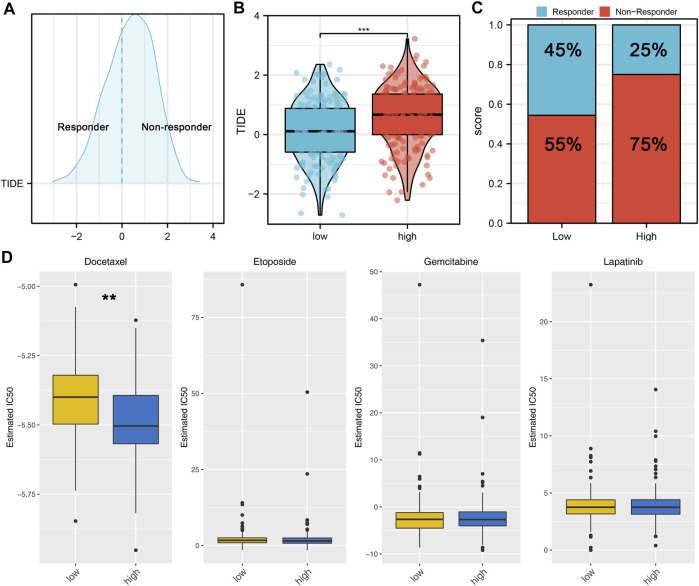
Risk score is associated with the sensibility of immunotherapy and chemotherapy. **(A)**: According to the TIDE analysis, the patients with the TIDE score < 0 were defined as responders, with the TIDE score > 0 were defined as nonresponders; **(B)**: Patients in a low-risk group might have a lower TIDE score than those in a high-risk group; **(C)**: Patients in a low-risk group might have a higher percentage of immunotherapy responders than those in a high-risk group; **(D)**: GDSC analysis was performed to evaluate the sensibility differences in docetaxel, etoposide, gemcitabine, and lapatinib.

Here, the CIBERSORT algorithm was applied to evaluate the composition of 22 immune cell types in the high- and low-risk groups. The correlation analysis of these 22 kinds of immune cells and risk score is shown in Figure 7A. The results revealed that macrophages (M1, M2), dendritic cells, monocytes, Tregs, and T regulatory cells were positively correlated with the risk scores. In contrast, the macrophages (M0), B memory cells, dendritic activated cells, CD4+ T cells, and NK cells were negatively correlated with the risk score (Figure 7B). Immuno score, stromal score, and estimate score were then calculated using the estimate package in R software. The results demonstrated that the stromal scores, immune score, and estimate score were positively correlated with the risk scores (Figures 7C,D). We next explored the underlying difference in the sensibility of immunotherapy and chemotherapy between high and low-risk patients. TIDE analysis was performed to assess the immunotherapy response rate of GC patients, in which the TIDE score < 0 was defined as responders and > 0 was defined as non-responders (Figure 8A). Meanwhile, we found that the GC patients in the low-risk group might have a lower TIDE score and a higher percentage of immunotherapy responders (Figures 8B,C). Moreover, we investigated the sensitivity difference in docetaxel, etoposide, gemcitabine, and lapatinib between high- and low-risk patients. The result showed that high-risk patients might be more sensitive to docetaxel (Figure 8D).

### AP000695.2 was Upregulated in Gastric Cancer and Promotes Cell Proliferation, Invasion, and Migration

**FIGURE 9:**
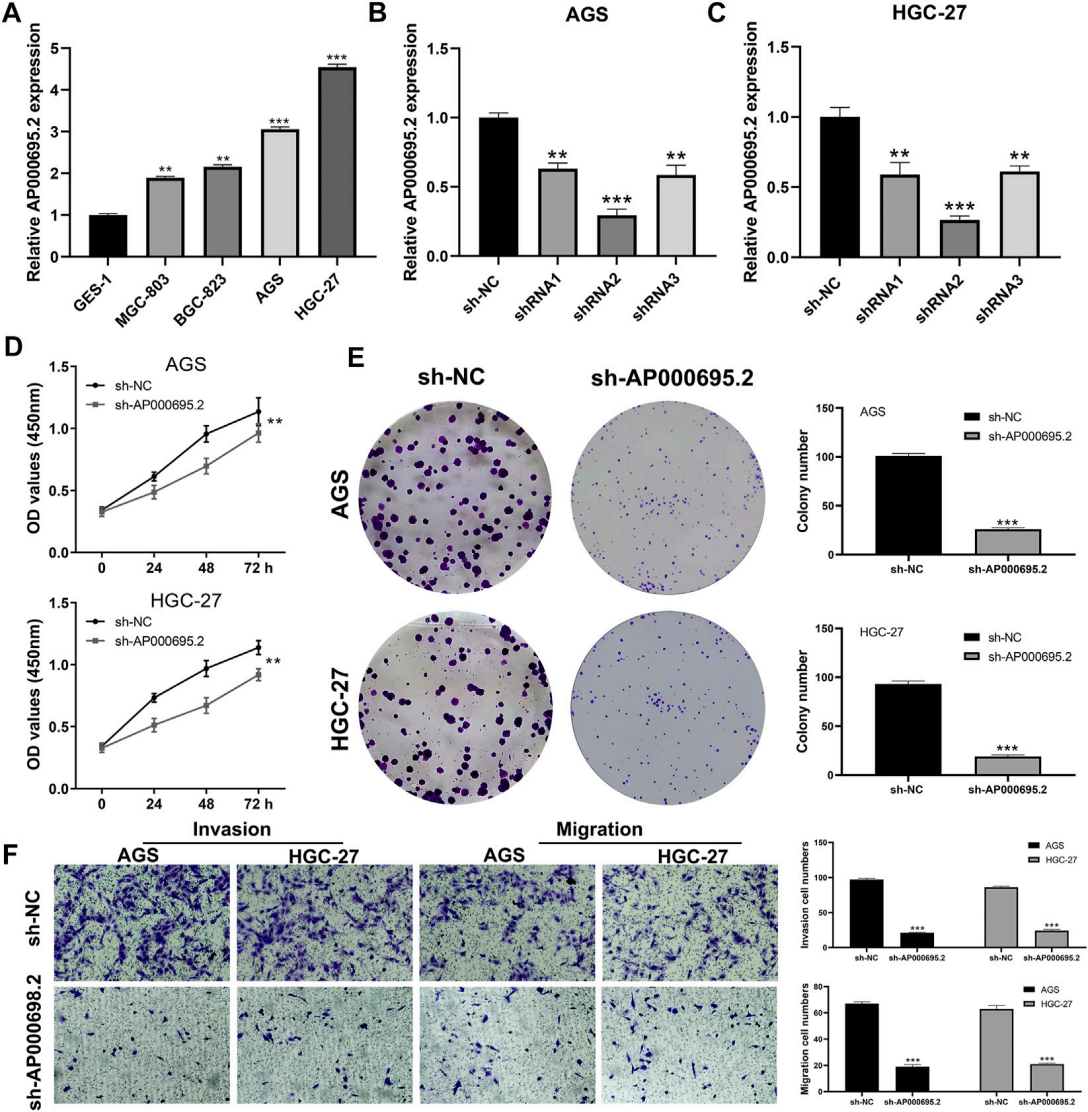
AP000695.2 was selected for further *in vitro* experiments. **(A)**: AP000695.2 was upregulated in GC cells; **(B,C)**: qRT-PCR result showed an satisfactory knockdown efficiency; **(D,E)**: CCK-8 and colony formation assays showed that the knockdown of AC000695.2 significantly inhibited GC cell proliferation; **(F)**: Transwell assay showed that the knockdown of AC000695.2 significantly inhibited GC cell invasion and migration.

Clinical correlation showed that AP000695.2 was associated with patients’ T and M stages, indicating that it could affect the biological function of GC. Therefore, lncRNA AP000695.2 was selected for further experiments. qRT-PCR showed that AP000695.2 was upregulated in GC cells compared with normal gastric mucosal cells (Figure 9A). AGS and HGC-27 were selected for AP000695.2 knockdown for their higher AP000695.2 expression. The result showed that shRNA#2 had the best knockdown efficiency, which was chosen for further experiments (Figures 9B,C). CCK-8 and colony formation assays indicated that the knockdown of AP000695.2 could significantly hamper the proliferation ability of GC cells (Figures 9D,E). Moreover, the transwell assay showed that the inhibition of AP000695.2 remarkably reduces the invasion and migration ability of GC cells (Figure 8F). The underlying biological mechanism of AP000695.2 in GC has also been explored (Supplementary Figure S1). The result showed that the pathway of epithelial–mesenchymal transition (EMT), interferon gamma response, TNF-α signaling *via* NKFB, inflammatory response, angiogenesis, and IL6/JAK/STAT3 signaling were significantly activated in the high AP000695.2 patients, indicating that AP000695.2 might exert its role through these pathways.

## Discussion

GC is the second leading cause of cancer-related deaths and the fourth most common cancer globally, which is of strong proliferation, metastasis, and invasion capabilities (Thrift and El-Serag, 2020). In this study, using the comprehensive bioinformatics analysis, we identified eight prognosis-related CMLs related to the progression and prognosis of OS. Meanwhile, our result showed that AP000695.2 could promote GC cell proliferation, invasion, and migration.

Immune checkpoints can induce long-lasting responses in many types of cancer, which encouraged oncologists to explore the potential treatments for the tumor with strong proliferation, metastasis, and invasion (Ross et al., 2009). However, currently, the significant response to immunotherapy is limited to a small number of patients, which highlights the need for more effective and new methods (Kroczek and Hamelmann, 2005). Many preclinical and clinical studies have been exploring the therapeutic potential of negative and positive costimulatory molecules in recent years. An in-depth understanding of the biological effects and molecular mechanisms on costimulatory molecules will undoubtedly help us understand the mechanism of cancer treatments targeting these molecules (Fu et al., 2020). By exploring CMLs in the TCGA-STAD cohort, we performed the differential expression analysis to obtain differentially expressed CMLs. Furthermore, we performed the LASSO Cox regression analysis, and univariate and multivariate Cox regression analyses to screen the prognostic-related CMLs. Considering the high heterogeneity of predictive prognosis with single genes, we constructed a prognostic prediction model with 8 CMLs, including AC027117.2, AC016737.1, LINC01614, AC147067.2, AP000695.2, LINC01094, HAGLR, and AL365181.3, which showed satisfactory predictive performance in the training group Moreover, univariate and multivariate analyses showed that eight CMLs-based risk scores are significantly associated with prognosis, independent with other clinical features. Taken together, these results proved the reliability and usefulness of the eight-CML-based prognostic prediction model. Meanwhile, we also explored the role of AP000695.2 in GC through *in vitro* experiments, and the result showed that AP000695.2 could promote the proliferation, invasion, and migration ability of GC cells.

Previous studies have identified specific signatures associated with patients’ prognoses in GC. For instance, Jiang et al. (2021) found that the eight ferroptosis-related molecules signature consisting TCFBR1, MYB, NFE2L2, ZFP36, TF, SLC1A5, NF2, and NOX4 could effectively indicate patients’ prognoses. The 1-, 3-, and 5-years AUC values were 0.654, 0.657, and 0.733 and 0.648, 0.623, and 0.629 in training and validation cohorts, respectively. Zhao et al. (2021) established a prognosis model based on 16 necroptosis-related lncRNAs, in which the 1-,2-, and 3-years AUC values were 0.754, 0.824, and 0.819 and 0.709, 0.701, and 0.713 in training and validation cohorts, respectively. In our study, we established a prognosis model consisting of eight prognosis-related CMLs AC027117.2, AC016737.1, LINC01614, AC147067.2, AP000695.2, LINC01094, HAGLR, and AL365181.3, which showed great prediction ability (the training cohort, 1-year AUC = 0.764, 3-year AUC = 0.810, 5-year AUC = 0.840; the validation cohort, 1-year AUC = 0.661, 3-year AUC = 0.718, 5-year AUC = 0.822). Meanwhile, we found that the low-risk patients might be more sensitive to immunotherapy, while high-risk patients might be more sensitive to docetaxel. This indicated that our model has the potential to guide the therapy option for GC patients.

Next, we try to explain the worse prognosis of high-risk patients in the biological mechanism. GSEA showed that EMT, KRAS signaling, and angiogenesis were aberrantly activated in high-risk patients. EMT is a process in which epithelial cells transdifferentiate to motile mesenchymal cells, which is essential for tumor cell movement and metastasis (Galluzzi et al., 2020). Li et al. (2019a) showed that tumor-associated neutrophils could induce EMT by IL-17a to facilitate migration and invasion in gastric cancer cells. Yue et al. (2019) indicated that ZMYM1 could bind to and mediate the repression of E-cadherin promoter by recruiting the CtBP/LSD1/CoREST complex, further promoting the EMT program and gastric metastasis. Moreover, Yang et al. (2013) revealed that the disorder of KRAS signaling and abnormal chromatin remodeling would lead to a poor prognosis and circulating tumor cell resistance to gastric cancer (Chen et al., 2021). Blood vessels in the tumor microenvironment are a key target for cancer therapeutic management (Viallard and Larrivée, 2017). Generally, angiogenesis plays an important role in tumor growth and metastasis. Unterleuthner et al. (2020) indicated that WNT2, derived from cancer-associated fibroblast, could increase tumor angiogenesis of colon cancer by upregulating ANG-2, IL-6, G-CSF, and PGF molecules, further resulting in the increased invasion and metastasis.

We further discovered that M2 macrophages, dendritic cells, monocytes, Tregs, and T regulatory cells were involved in the high-risk group, compared with the low-risk group. M2 macrophages have been reported to facilitate tumor progression in multiple cancers (Mantovani et al., 2002). Cao et al. (2019) showed that lncRNA MM2P could induce M2 macrophage polarization in a STAT6 phosphorylation-depend way, further promoting tumorigenesis, tumor growth, and angiogenesis. In gastric cancer, Li et al. (2019b) revealed that gastric cancer–derived mesenchymal stromal cells trigger M2 macrophage polarization that facilitates metastasis and EMT. Studies have shown that low levels of endogenous IL23 produced by tumor-associated macrophages, dendritic cells, or tumor cells may promote inflammation, which is conducive to the occurrence and progression of early tumors (Neurath, 2019). In addition, our analysis found that high immune scores are associated with low OS, which indicates that favorable factors such as certain cytokines, chemokines, and immune cell subunits play an essential role in tumor immunity.

In summary, this study evaluated the role of CMLs in GC and constructed a CML-based prognostic predictive model, which showed satisfactory prediction efficiency. Univariate and multivariate independent prognostic analyses showed that eight CML-based risk scores are significantly and independently associated with prognosis. Meanwhile, some oncogenic signaling pathways were abnormally activated in high-risk patients, including EMT, KRAS signaling, and angiogenesis. Moreover, we also found a significant difference in the immune microenvironment between high- and low-risk patients. Finally, lncRNA AP000659.2 was selected for further experiments. *In vitro* results showed that AP000695.2 promotes GC proliferation, invasion, and migration.

## Data Availability

Publicly available datasets were analyzed in this study. These data can be found here: https://portal.gdc.cancer.gov/.

## Supplementary Material

Supplementary Figure S1 GSEA of high and low AP000695.2 patients.

## References

[B1] Bluestone J. A. (1995). New Perspectives of C1328-137-Mediated T Cell Costimulation. Immunity 2 (6), 555–559. 10.1016/1074-7613(95)90000-4 7540940

[B2] Cao J. Dong R. Jiang L. Gong Y. Yuan M. You J. (2019). LncRNA-MM2P Identified as a Modulator of Macrophage M2 Polarization. Cancer Immunol. Res. 7 (2), 292–305. 10.1158/2326-6066.Cir-18-0145 30459152

[B3] Chen B. Khodadoust M. S. Liu C. L. Newman A. M. Alizadeh A. A. (2018). Profiling Tumor Infiltrating Immune Cells with CIBERSORT. Methods Mol. Biol. 1711, 243–259. 10.1007/978-1-4939-7493-1_12 29344893 PMC5895181

[B4] Chen Y. Li Y. Qi C. Zhang C. Liu D. Deng Y. (2021). Dysregulated KRAS Gene-Signaling axis and Abnormal Chromatin Remodeling Drive Therapeutic Resistance in Heterogeneous-Sized Circulating Tumor Cells in Gastric Cancer Patients. Cancer Lett. 517, 78–87. 10.1016/j.canlet.2021.06.002 34126192

[B5] Correa P. (2013). Gastric Cancer. Gastroenterology Clin. N. Am. 42 (2), 211–217. 10.1016/j.gtc.2013.01.002 PMC3995345 23639637

[B6] Croft M. Benedict C. A. Ware C. F. (2013). Clinical Targeting of the TNF and TNFR Superfamilies. Nat. Rev. Drug Discov. 12 (2), 147–168. 10.1038/nrd3930 23334208 PMC3625401

[B7] Ferrè F. Colantoni A. Helmer-Citterich M. (2016). Revealing Protein-lncRNA Interaction. Brief. Bioinform 17 (1), 106–116. 10.1093/bib/bbv031 26041786 PMC4719072

[B8] Fu Y. Lin Q. Zhang Z. Zhang L. (2020). Therapeutic Strategies for the Costimulatory Molecule OX40 in T-Cell-Mediated Immunity. Acta Pharm. Sin. B 10 (3), 414–433. 10.1016/j.apsb.2019.08.010 32140389 PMC7049610

[B9] Galluzzi L. Humeau J. Buqué A. Zitvogel L. Kroemer G. (2020). Immunostimulation with Chemotherapy in the Era of Immune Checkpoint Inhibitors. Nat. Rev. Clin. Oncol. 17 (12), 725–741. 10.1038/s41571-020-0413-z 32760014

[B10] Jiang P. Gu S. Pan D. Fu J. Sahu A. Hu X. (2018). Signatures of T Cell Dysfunction and Exclusion Predict Cancer Immunotherapy Response. Nat. Med. 24 (10), 1550–1558. 10.1038/s41591-018-0136-1 30127393 PMC6487502

[B11] Jiang X. Yan Q. Xie L. Xu S. Jiang K. Huang J. (2021). Construction and Validation of a Ferroptosis-Related Prognostic Model for Gastric Cancer. J. Oncol. 2021, 1–14. 10.1155/2021/6635526 PMC7937463 33727924

[B12] Karimi M. H. Ebadi P. Pourfathollah A. A. (2013). Association of Cytokine/costimulatory Molecule Polymorphism and Allograft Rejection: a Comparative Review. Expert Rev. Clin. Immunol. 9 (11), 1099–1112. 10.1586/1744666x.2013.844462 24168415

[B13] Kroczek R. Hamelmann E. (2005). T-Cell Costimulatory Molecules: Optimal Targets for the Treatment of Allergic Airway Disease with Monoclonal Antibodies. J. Allergy Clin. Immunol. 116 (4), 906–909. 10.1016/j.jaci.2005.07.005 16210068

[B14] Li S. Cong X. Gao H. Lan X. Li Z. Wang W. (2019a). Tumor-associated Neutrophils Induce EMT by IL-17a to Promote Migration and Invasion in Gastric Cancer Cells. J. Exp. Clin. Cancer Res. 38 (1), 6. 10.1186/s13046-018-1003-0 30616627 PMC6323742

[B15] Li W. Zhang X. Wu F. Zhou Y. Bao Z. Li H. (2019b). Gastric Cancer-Derived Mesenchymal Stromal Cells Trigger M2 Macrophage Polarization that Promotes Metastasis and EMT in Gastric Cancer. Cell Death Dis. 10 (12), 918. 10.1038/s41419-019-2131-y 31801938 PMC6892854

[B16] Mantovani A. Sozzani S. Locati M. Allavena P. Sica A. (2002). Macrophage Polarization: Tumor-Associated Macrophages as a Paradigm for Polarized M2 Mononuclear Phagocytes. Trends Immunol. 23 (11), 549–555. 10.1016/s1471-4906(02)02302-5 12401408

[B17] Mueller D. L. (2000). T Cells: A Proliferation of Costimulatory Molecules. Curr. Biol. 10 (6), R227–R230. 10.1016/s0960-9822(00)00400-0 10744968

[B18] Neurath M. F. (2019). IL-23 in Inflammatory Bowel Diseases and Colon Cancer. Cytokine & Growth Factor Rev. 45, 1–8. 10.1016/j.cytogfr.2018.12.002 30563755

[B19] Pasquini S. Xiang Z. Wang Y. He Z. Deng H. Blaszczyk-Thurin M. (1997). Cytokines and Costimulatory Molecules as Genetic Adjuvants. Immunol. Cell Biol. 75 (4), 397–401. 10.1038/icb.1997.62 9315484

[B20] Podojil J. R. Miller S. D. (2009). Molecular Mechanisms of T-Cell Receptor and Costimulatory Molecule Ligation/blockade in Autoimmune Disease Therapy. Immunol. Rev. 229 (1), 337–355. 10.1111/j.1600-065X.2009.00773.x 19426232 PMC2845642

[B21] Ross A. C. Chen Q. Ma Y. (2009). Augmentation of Antibody Responses by Retinoic Acid and Costimulatory Molecules. Seminars Immunol. 21 (1), 42–50. 10.1016/j.smim.2008.08.004 PMC2615053 18819820

[B22] Schietinger A. Greenberg P. D. (2014). Tolerance and Exhaustion: Defining Mechanisms of T Cell Dysfunction. Trends Immunol. 35 (2), 51–60. 10.1016/j.it.2013.10.001 24210163 PMC3946600

[B23] Schildberg F. A. Klein S. R. Freeman G. J. Sharpe A. H. (2016). Coinhibitory Pathways in the B7-CD28 Ligand-Receptor Family. Immunity 44 (5), 955–972. 10.1016/j.immuni.2016.05.002 27192563 PMC4905708

[B24] Sitarz R. Skierucha M. Mielko J. Offerhaus J. Maciejewski R. Polkowski W. (2018). Gastric Cancer: Epidemiology, Prevention, Classification, and Treatment. Cmar 10, 239–248. 10.2147/cmar.S149619 PMC5808709 29445300

[B25] Smyth E. C. Nilsson M. Grabsch H. I. van Grieken N. C. Lordick F. (2020). Gastric Cancer. Lancet 396 (10251), 635–648. 10.1016/s0140-6736(20)31288-5 32861308

[B26] Song Z. Wu Y. Yang J. Yang D. Fang X. (2017). Progress in the Treatment of Advanced Gastric Cancer. Tumour Biol. 39 (7), 101042831771462. 10.1177/1010428317714626 28671042

[B27] Stewart O. A. Wu F. Chen Y. (2020). The Role of Gastric Microbiota in Gastric Cancer. Gut Microbes 11 (5), 1220–1230. 10.1080/19490976.2020.1762520 32449430 PMC7524314

[B28] Subramanian A. Tamayo P. Mootha V. K. Mukherjee S. Ebert B. L. Gillette M. A. (2005). Gene Set Enrichment Analysis: a Knowledge-Based Approach for Interpreting Genome-wide Expression Profiles. Proc. Natl. Acad. Sci. U.S.A. 102 (43), 15545–15550. 10.1073/pnas.0506580102 16199517 PMC1239896

[B29] Tamada K. Chen L. (2000). T Lymphocyte Costimulatory Molecules in Host Defense and Immunologic Diseases. Ann. Allergy, Asthma & Immunol. 85 (3), 164–176. quiz 175-167. 10.1016/s1081-1206(10)62462-3 11030270

[B30] Thrift A. P. El-Serag H. B. (2020). Burden of Gastric Cancer. Clin. Gastroenterology Hepatology 18 (3), 534–542. 10.1016/j.cgh.2019.07.045 PMC8859863 31362118

[B31] Unterleuthner D. Neuhold P. Schwarz K. Janker L. Neuditschko B. Nivarthi H. (2020). Cancer-associated Fibroblast-Derived WNT2 Increases Tumor Angiogenesis in Colon Cancer. Angiogenesis 23 (2), 159–177. 10.1007/s10456-019-09688-8 31667643 PMC7160098

[B32] Viallard C. Larrivée B. (2017). Tumor Angiogenesis and Vascular Normalization: Alternative Therapeutic Targets. Angiogenesis 20 (4), 409–426. 10.1007/s10456-017-9562-9 28660302

[B33] Wei L. Sun J. Zhang N. Zheng Y. Wang X. Lv L. (2020). Noncoding RNAs in Gastric Cancer: Implications for Drug Resistance. Mol. Cancer 19 (1), 62. 10.1186/s12943-020-01185-7 32192494 PMC7081551

[B34] Wei S. C. Duffy C. R. Allison J. P. (2018). Fundamental Mechanisms of Immune Checkpoint Blockade Therapy. Cancer Discov. 8 (9), 1069–1086. 10.1158/2159-8290.Cd-18-0367 30115704

[B35] Yang W. Soares J. Greninger P. Edelman E. J. Lightfoot H. Forbes S. (2013). Genomics of Drug Sensitivity in Cancer (GDSC): a Resource for Therapeutic Biomarker Discovery in Cancer Cells. Nucleic Acids Res. 41, D955–D961. Database issue). 10.1093/nar/gks1111 23180760 PMC3531057

[B36] Yu G. Wang L.-G. Han Y. He Q.-Y. (2012). clusterProfiler: an R Package for Comparing Biological Themes Among Gene Clusters. OMICS A J. Integr. Biol. 16 (5), 284–287. 10.1089/omi.2011.0118 PMC3339379 22455463

[B37] Yue B. Song C. Yang L. Cui R. Cheng X. Zhang Z. (2019). METTL3-mediated N6-Methyladenosine Modification Is Critical for Epithelial-Mesenchymal Transition and Metastasis of Gastric Cancer. Mol. Cancer 18 (1), 142. 10.1186/s12943-019-1065-4 31607270 PMC6790244

[B38] Zhang M. Wang N. Song P. Fu Y. Ren Y. Li Z. (2020). LncRNA GATA3‐AS1 Facilitates Tumour Progression and Immune Escape in Triple‐negative Breast Cancer through Destabilization of GATA3 but Stabilization of PD‐L1. Cell Prolif. 53 (9), e12855. 10.1111/cpr.12855 32687248 PMC7507373

[B39] Zhao L. Liu Y. Zhang J. Liu Y. Qi Q. (2019). LncRNA SNHG14/miR-5590-3p/ZEB1 Positive Feedback Loop Promoted Diffuse Large B Cell Lymphoma Progression and Immune Evasion through Regulating PD-1/pd-L1 Checkpoint. Cell Death Dis. 10 (10), 731. 10.1038/s41419-019-1886-5 31570691 PMC6769008

[B40] Zhao Z. Liu H. Zhou X. Fang D. Ou X. Ye J. (2021). Necroptosis-Related lncRNAs: Predicting Prognosis and the Distinction between the Cold and Hot Tumors in Gastric Cancer. J. Oncol. 2021, 1–16. 10.1155/2021/6718443 PMC8592775 34790235

