# Supplementary figures and images for: Identification of Costimulatory Molecule–Related lncRNAs Associated With Gastric Carcinoma Progression: Evidence From Bioinformatics Analysis and Cell Experiments
Source: Front Genet. 2022 Aug 5;13:950222. doi: 10.3389/fgene.2022.950222 (PMC9388737; doi:10.3389/fgene.2022.950222)

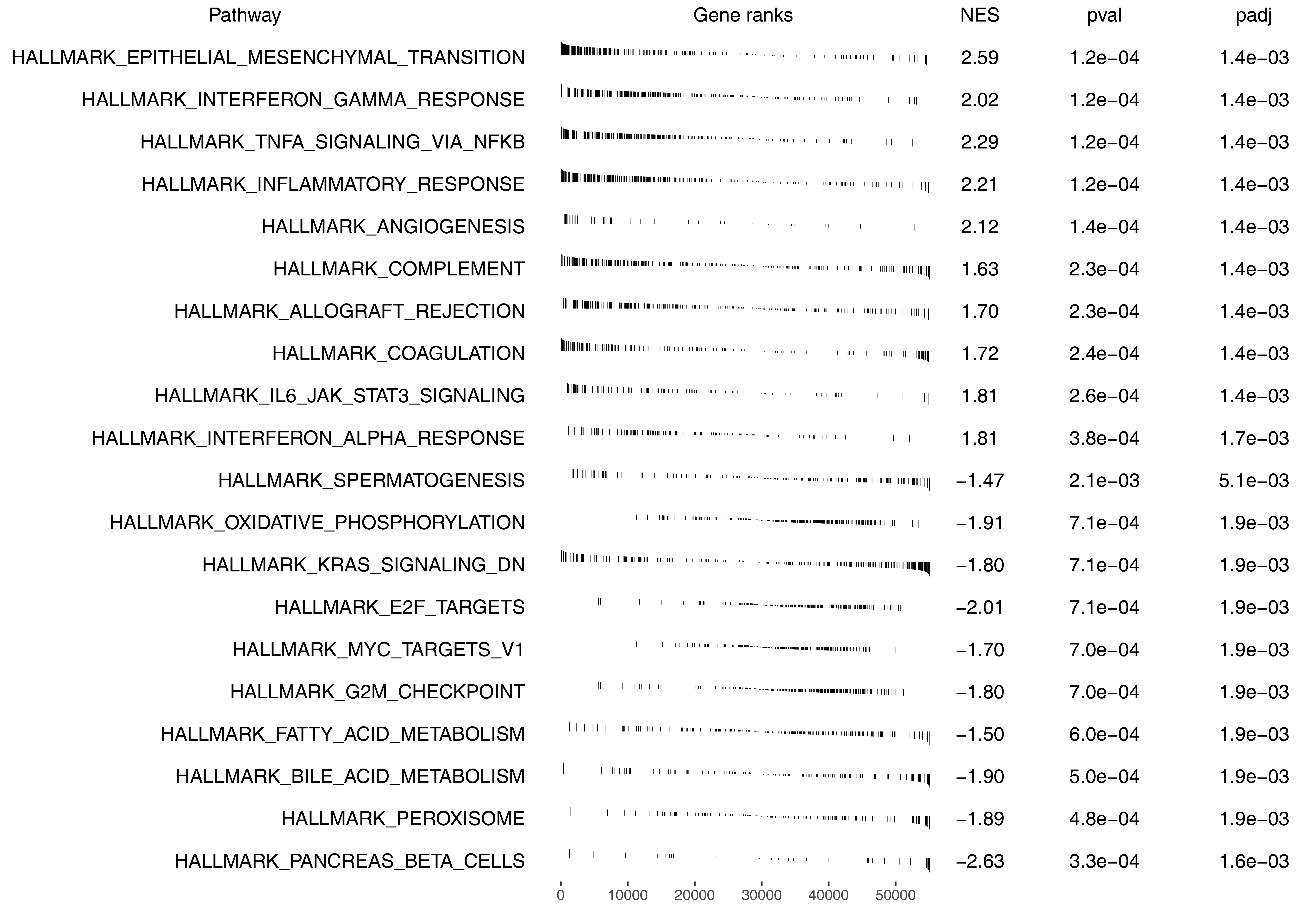

Supplement: Supplementary file 1 [file Image1.tif]
